# Dynamic changes in quality of life in older patients with chronic obstructive pulmonary disease: a 7-year follow up

**DOI:** 10.1186/s12955-024-02296-1

**Published:** 2024-09-11

**Authors:** Chun-Hsiang Yu, Sheng-Han Tsai, Jo-Ying Hung, Pei-Fang Su, Chih-Hui Hsu, Xin-Min Liao, Tzuen-Ren Hsiue, Chiung-Zuei Chen

**Affiliations:** 1 Division of Pulmonary Medicine, Department of Internal Medicine, National Cheng Kung University Hospital, College of Medicine, National Cheng Kung University, Tainan, Taiwan; 2 Division of General Medicine, Department of Internal Medicine, College of Medicine, National Cheng Kung University Hospital, National Cheng Kung University, Tainan, Taiwan; 3 Department of Statistics, National Cheng Kung University, Tainan, Taiwan; 4 Clinical Medicine Research Center, National Cheng Kung University Hospital, Tainan, Taiwan

**Keywords:** Chronic obstructive pulmonary disease, Quality of life, Dynamic change.

## Abstract

**Purposes:**

: Chronic obstructive pulmonary disease (COPD) is a major cause of the rapid decline of health-related quality of life (HRQoL), associated with accelerated frailty in older populations. This study aimed to analyse the long-term dynamic changes of HRQoL and the predictive factors for the rapid decline of HRQoL in older patients with COPD.

**Methods:**

Overall 244 patients with COPD, aged ≧ 65 years from one medical centre were enrolled between March 2012 and July 2020. Further, we prospectively assessed HRQoL scores with utility values, using EuroQol Five-Dimension (EQ-5D) questionnaires. Additionally, long-term dynamic changes in HRQoL were analysed using the Kernel smoothing method and examined the factors contributing to the deterioration of HRQoL using a linear mixed effects model.

**Results:**

Older patients with COPD with forced expiration volume (FEV1) < 50% of prediction entered the phase of rapid and continuous decline of HRQoL ~ 2 years after enrolment, but patients with FEV1 ≥ 50% of prediction without rapidly declined HRQoL during 7 years follow up. Therefore, FEV1 < 50% of prediction is a novel predictor for the rapid decline of HRQoL. The course of rapidly declining HRQoL occurred, initially in the usual activities and pain/discomfort domains, followed by the morbidity, self-care, and depression/anxiety domains ~ 2 and 4 years after enrolment, respectively. The mixed effects model indicated that both FEV1 < 50% of prediction and a history of severe acute exacerbation (SAE) requiring hospitalisation were contributing factors for deterioration in HRQoL .

**Conclusions:**

Both FEV1 < 50% of prediction and exacerbations requiring hospitalisation were contributing factors for the deterioration of HRQoL in long-term follow up. Additionally, FEV1 < 50% of prediction was a novel predictor for patients entering the phase of rapid decline of HRQoL.

**Supplementary Information:**

The online version contains supplementary material available at 10.1186/s12955-024-02296-1.

## Introduction

Chronic obstructive pulmonary disease (COPD) is a critical cause of disability, morbidity, and mortality globally [1]. Burden of COPD healthcare costs is a universal issue. As the older population grows worldwide, it is predicted that COPD prevalence will increase due to difficulties in eliminating risk factors such as cigarette smoking [2]. 

Successful ageing represents a good quality of life in old age [3]. However, patients with COPD suffer from poor mental and physical functions because of the disease and associated disability [4]. Impaired health-related quality of life (HRQoL) is especially prevalent among older patients with COPD [5]. Previous studies have revealed the negative impacts of COPD on physical and psychological health. The determining factors influencing the quality of life for patients with COPD have been investigated using various short-term analyses [6, 7]. The life trajectory of COPD has traditionally been illustrated as a continuous accelerated decay in health status accompanied by frequent hospital admissions and exacerbations over time [8]. However, the dynamic changes in HRQoL and predictive factors for rapidly deteriorating HRQoL during long-term follow up for older patients with COPD remain unknown. This study aimed to analyse the dynamic changes in HRQoL for older patients with COPD and their predictive factors for continuous and rapidly declining health status. Therefore, timely and appropriate intervention may improve the quality of life for older individuals with COPD.

## Methods

### Patients

This retrospective study enrolled patients with COPD, aged ≧ 65 years from the outpatient clinics of National Cheng Kung University Hospital (NCKUH) from 1 March 2012 to 31 July2020. COPD was diagnosed according to the diagnosis guidelines and criteria [9]. Patients were divided into mild (forced expiration volume [FEV1] ≥ 80% of prediction), moderate (FEV1 ≥ 50% but < 80% of prediction), and severe (FEV1 < 50% of prediction) groups of pulmonary function stages. Furthermore, demographic data including age, sex, body mass index, education, smoking status, comorbidity, medication, and history of severe acute exacerbation (SAE) in the preceding year were recorded.

### Dynamic changes in health-related quality of life

The HRQoL of the enrolled patients was consistently monitored with the 3-Level version of the European Quality of Life-5 Dimensions (EQ-5D-3 L). Validated Taiwanese versions of EQ-5D-3 L questionnaires were used in this study [10]. The EQ-5D-3 L questionnaires were administered by the COPD case manager at the outpatient department. Serial measurement of the EQ-5D score was analysed for the trend of dynamic change of HRQoL. Written informed consent was obtained from all participants, which must be reviewed and approved by the ethics committee, prior to enrolment in this study.

Severe acute respiratory syndrome coronavirus 2 was confirmed to have spread to Taiwan on 21 January 2020, and the COVID-19 (coronavirus disease 2019) pandemic period overlapped the period of our study by approximately 6 months. Therefore, we also repeated our analysis for the dynamic change of HRQoL in the period between 1 March 2012 and 31 December 2019 to avoid the effect of COVID-19 on the result of this study.

### Statistical analysis

Patient characteristics for the cohort were presented in the form of descriptive statistics, with mean ± standard deviation for continuous variables and sample size (percentage) for categorical variables. As for statistical comparisons among groups, continuous variables were analysed using the t-test, and categorical variables were analysed using the chi-square test. Each test of significance was two-sided, with the significance level set at 0.05.

When analysing the factors influencing the utility value of quality of life with EQ-5D, we employed a repeated-measures mixed-effects model to account for random effects of the same subject measured at different times during the longitudinal follow-up. The fixed-effect variables included in the construction of the models were as follows: pulmonary function test, age, gender, the presence of previous SAE, the presence of comorbidities, and educational level.

A linear regression model was fitted between the utility score and the time-point of mild, moderate and severe groups of COPD to predict how the utility score decreased over time. The dynamic change of HRQoL was analysed using a kernel smoothing method, with a locally weighted scatterplot smoothing (LOESS) curve fitted for the utility score. All analyses were performed using R software (R version 4.2.1 statistical software, R Core Team (2021). R: A language and environment for statistical computing. R Foundation for Statistical Computing, Vienna, Austria. URL https://www.R-project.org/).

## Results

In this study, 244 older patients with COPD were enrolled from 1 March 2012 to 31 July 2020; the average follow-up time was 30 months in this study. Of these, 155 participants (63.5%) have at least two recordings for utility values, for a total of 804 repeated measurements. The average number of responses to questionnaire assessments was 3.3 times. The mean age of participants was 72 years, and 94% of participants were male. The severity of pulmonary function stages was associated with patients having SAEs in the preceding one-year, a lower body mass index (BMI) and a modified Medical Research Council (mMRC) dyspnoea score, a higher ratio of heart failure and mortality, and using triple therapy (Table 1).

**Table 1:** Demographic data for older patients with COPD

	Pulmonary function stages			*p*-value
	Mild (FEV1 > 80%) (*N* = 73)	Moderate (FEV1 50–80%) (*N* = 116)	Severe (FEV1 < 50%) (*N* = 55)	
Variables	*n* (%)/mean ± SD	*n* (%)/mean ± SD	*n* (%)/mean ± SD	
Age	72.1 ± 4.5	72.4 ± 4.0	72.0 ± 4.2	0.854
Male	70 (95.89)	111 (95.69)	50 (90.91)	0.376
Acute exacerbation last year	10 (13.70)	25 (21.55)	19 (34.55)	0.020
Smoking status^†^				
Never smoking	6 (8.96)	11 (10.00)	4 (7.41)	0.538
Quit smoking	43 (64.18)	69 (62.73)	41 (75.93)	
Current smoker	18 (26.87)	30 (27.27)	9 (16.67)	
Cigarettes (Pack-year^†^)	49.5 ± 37.1	56.2 ± 36.6	54.9 ± 34.0	0.496
BMI^†^	23.98 ± 3.55	24.64 ± 4.26	21.71 ± 3.23	< 0.001
mMRC^†^	1.55 ± 0.76	1.98 ± 0.73	2.73 ± 0.83	< 0.001
FEV1%^†^	91.49 ± 10.32	65.13 ± 8.96	37.96 ± 6.85	< 0.001
Comorbidity				
Stroke	2 (2.74)	2 (1.72)	2 (3.64)	0.663
CAD	11 (15.07)	9 (7.76)	8 (14.55)	0.221
Heart failure	3 (4.11)	13 (11.21)	11 (20.00)	0.018
CKD	4 (5.48)	12 (10.34)	4 (7.27)	0.475
Cirrhosis	1 (1.37)	3 (2.59)	0 (0.00)	0.814
Cancer	2 (2.74)	9 (7.76)	7 (12.73)	0.099
Education^†^				
None	3 (6.12)	8 (9.20)	4 (11.76)	0.921
Elementary school	23 (31.51)	45 (51.72)	18 (32.73)	
High school	14 (19.18)	23 (26.44)	8 (14.55)	
College	9 (12.33)	11 (12.64)	4 (7.27)	
Mortality^†^	1 (2.00)	9 (9.68)	12 (24.00)	0.002
Inhaled therapy				
LAMA	20 (27.40)	34 (29.31)	7 (12.73)	0.055
LAMA + LABA	16 (21.92)	46 (39.66)	14 (25.45)	0.022
LABA + ICS	17 (23.29)	13 (11.21)	13 (23.64)	0.043
Triple therapy	5 (6.85)	9 (7.76)	16 (29.09)	< 0.001

^†^Smoking status had 13 missing values. Pack year had 33 missing values. BMI had 1 missing value. MMRC had 2 missing values. FEV1% had 20 missing values. Education had 74 missing values. Mortality had 51 missing values. COPD: Chronic Obstructive Pulmonary Disease; FEV1: Forced Expiratory Volume in the First second; SD: Standard Deviation; BMI: Body Mass Index; mMRC: modified Medical Research Council; CAD: Coronary Artery Disease; CKD: Chronic Kidney Disease; LAMA: Long-Acting Muscarinic Antagonist; LABA: Long-Acting Beta-Agonist; ICS: Inhaled Corticosteroids

### Dynamic HRQoL change evaluated by EQ-5D utility according to the severity of pulmonary function stages

**Fig. 1:**
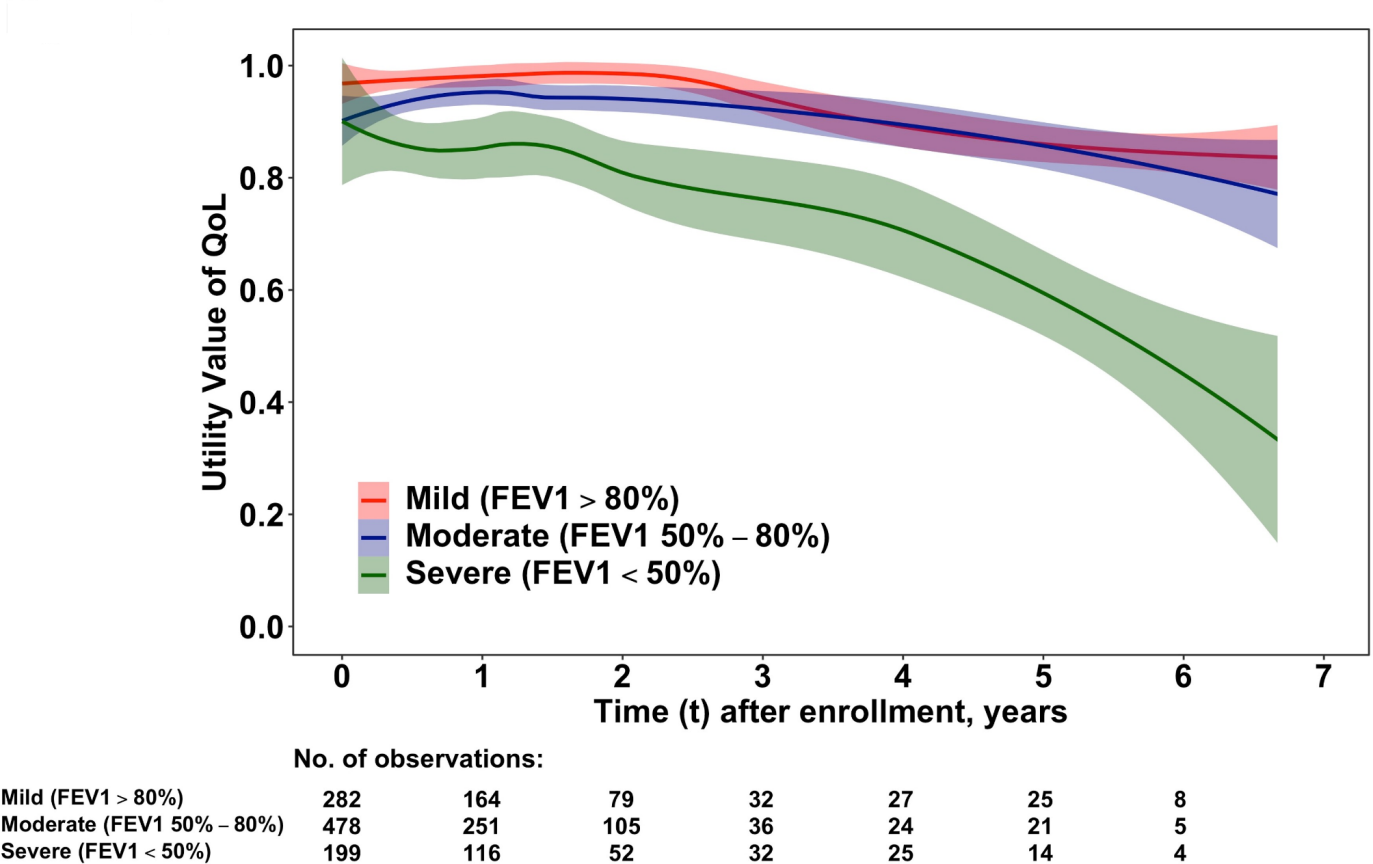
Dynamic changes of utility values of quality of life (QoL) in older COPD patients with different stages of pulmonary function. The dynamic change of utility values of EQ-5D-3 L was analysed by a kernel smoothing method. Patients with FEV1 < 50% of prediction entered the phase of rapid declined of utility values approximately 2 years later and a more rapid decline was started approximately 4 years later from the beginning of follow-up

Figure 1 describes fluctuations in utility values of EQ-5D-3 L over time. A noticeable difference can be observed in utility values between the severe and mild/moderate pulmonary function stages during the 7-year follow up. For the first 2 years, no noticeable dynamic changes were displayed in the utility scores of the severe group. However, a decrease in the utility score was observed in the severe group 2 years later, and a noticeably accelerated decrease in the utility score of the severe group was observed 4 years later.

**Fig. 2:**
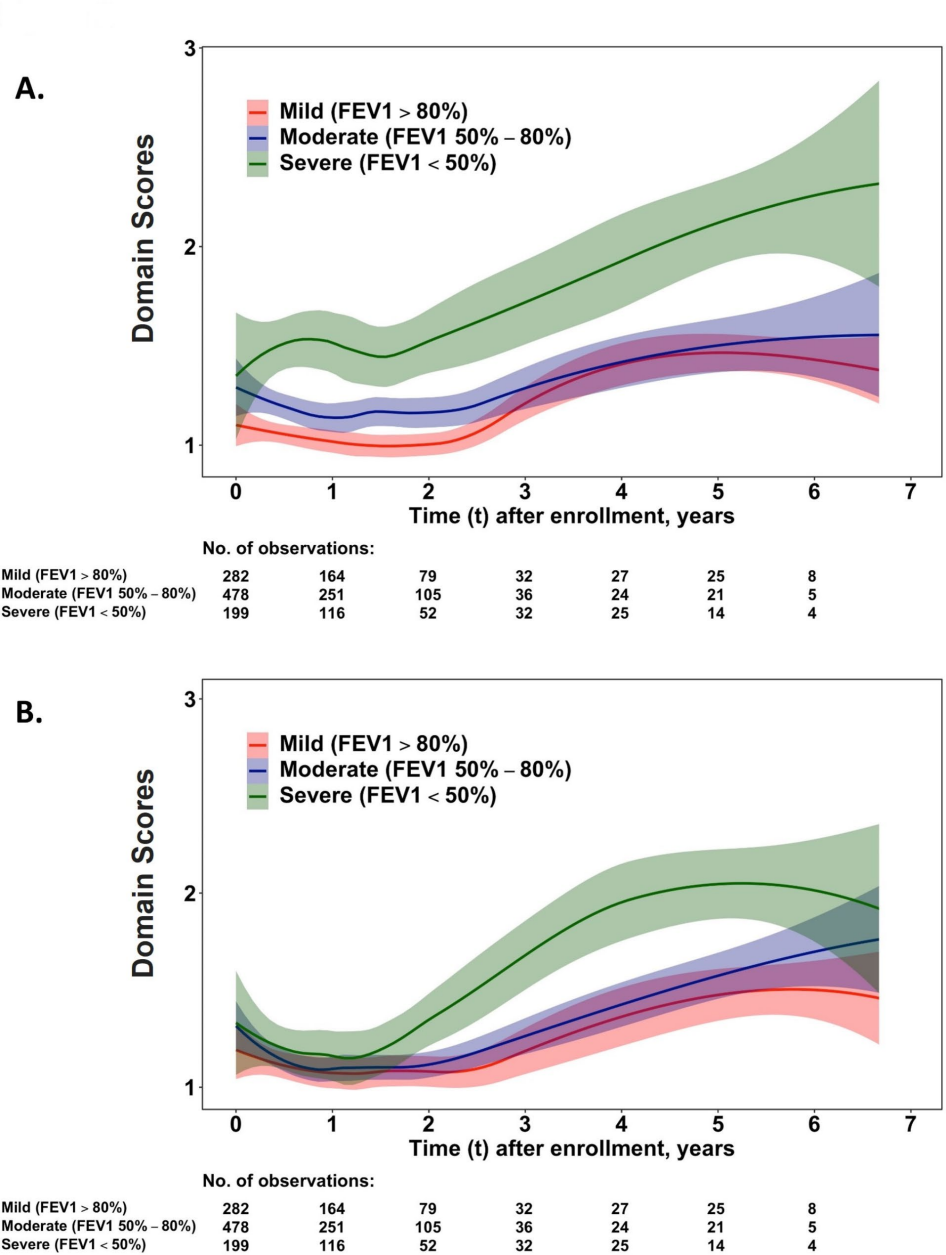
Dynamic changes of health domains in ‘usual activities’ **(A)** and ‘pain/discomfort’ **(B)** for older COPD patients with different stages of pulmonary function. The dynamic change of domain scores of EQ-5D-3 L was analysed by a kernel smoothing method. Distinctly worsened scores in patients with FEV1 < 50% of prediction were noticed at approximately 2 years later from the beginning of follow-up in the **(A)** ‘usual activity’ and **(B)** ‘pain/discomfort’ domains. The dynamic change of ‘pain/discomfort’ domain reached a plateau approximately 4 years later from the beginning of follow-up, but continuous rapid progression of domain scores in the ‘usual activity’ domain

**Fig. 3:**
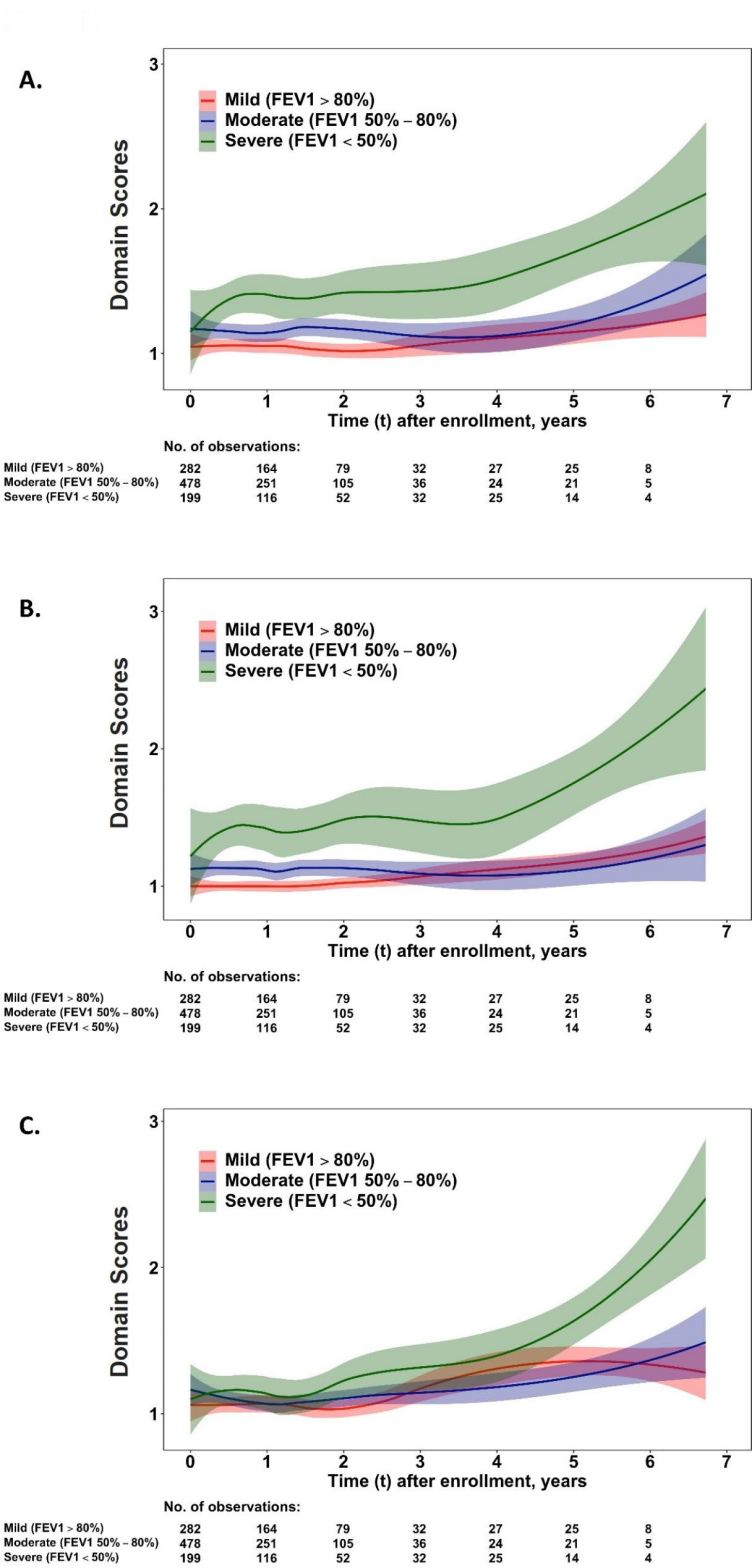
Dynamic changes of health domains in ‘mobility’ **(A)**, ‘self-care’ **(B)**, and ‘anxiety/depression’ **(C)** for older COPD patients with different stages of pulmonary function. The dynamic change of domain scores of EQ-5D-3 L was analysed by a kernel smoothing method. Distinctly worsened scores in patients with FEV1 < 50% of prediction were noticed at approximately 4 years later from the beginning of follow-up in the **(A)** ‘mobility’, **(B)** ‘self-care’ and **(C)** ‘anxiety/depression’ domains

Figures 2 and 3 describe the dynamic change in HRQoL on all five domains of the EQ-5D-3 L. In the domain of ‘usual activities’ and ‘pain/discomfort’, increased scores in the group of severe pulmonary function stage were observed after 2 years (Fig. 2A and B). After 4 years of disease tracking, a plateau in the ‘pain/discomfort’ dimension was observed in the group of severe pulmonary function stage, but limitations in ‘usual activities’ continued to worsen during follow up. For participants in the mild and moderate severity groups, scores worsened in ‘usual activities’ and ‘pain/discomfort’ after following up for 2 years, but these scores were significantly lower compared to the severe group (Fig. 2).

In Fig. 3, the rapid HRQoL deterioration in ‘mobility’, ‘self-care’, and ‘depression/anxiety’ emerged following declines in the ‘usual activity’ and ‘pain/discomfort’ domains after disease surveillance for 4 years (Fig. 3A, B, and C).

For the dynamic score changes in the five domains of the EQ-5D, the scores in ‘usual activities’, ‘mobility’, and ‘self-care’ (Figs. 2A and 3A, and 3B) were markedly worse in the group of severe pulmonary function stage than in the mild/moderate groups from the beginning of follow-up. Comparatively, clearly separated curves were illustrated for the ‘pain/discomfort’ domain 2 years later (Fig. 2B) andthe ‘anxiety/depression’ domain 5 years later (Fig. 3C).

Using a mixed effects model (Table 2), both FEV1 < 50% of prediction and SAE requiring hospitalisation (SAE) history were contributing factors for the deterioration of HRQoL during the 7-year follow up. Regarding the participants’ comorbidities, patients with COPD and chronic kidney disease (CKD) suffered from worse HRQoL than other comorbidities, according to the results of the mixed effects model.

**Table 2:** Mixed effects model of factors associated with eq. 5D utility

	Mixed effects model	
Variables	β ± SE	*p*-value
Pulmonary function stages		
Mild	Ref.	
Moderate	-0.03 ± 0.02	0.179
Severe	-0.10 ± 0.03	< 0.001
Age	-0.0002 ± 0.002	0.933
Gender		
Female	Ref.	
Male	0.0004 ± 0.04	0.992
SAE history		
No	Ref.	
Yes	-0.09 ± 0.02	< 0.001
Comorbidity		
Stroke	-0.01 ± 0.08	0.926
CAD	-0.08 ± 0.05	0.102
Heart failure	0.06 ± 0.03	0.079
CKD	-0.12 ± 0.04	0.005
Cirrhosis	-0.10 ± 0.08	0.174
Cancer	-0.001 ± 0.05	0.975
Education		
None	Ref.	
Elementary school	0.06 ± 0.04	0.092
High School	0.05 ± 0.04	0.154
College	0.06 ± 0.04	0.121

SE: Standard Error, SAE: Severe Acute Exacerbation, CAD: Coronary Artery Disease, CKD: Chronic Kidney Disease

Because of concerns over the effect of COVID-19 on the result, repeated analysis was performed for the dynamic change of HRQoL. Supplementary Figs. 1, 2, and 3 showed similar results for dynamic change of HRQoL in utility and five domains of the Eq. 5-D 3 L in the period between 1 March 2012 and 31 December 2019 compared to Figs. 1 and 2, and 3.

## Discussion

The dynamic changes in HRQoL for older patients with COPD have not been explored previously. This study found that older patients with COPD with an FEV1 < 50% of prediction was a novel predictive factor for the dynamic deterioration of HRQoL in the 7-year follow up. Patients with an FEV1 < 50% of prediction have a prolonged stable phase for ~ 2 years with preserved HRQoL, and then they progress to the phase of rapid decline of HRQoL deterioration. Deteriorating HRQoL began after 2 years of follow up, and rapid decline was noticed after 4 years of follow-up. In a study by Antonelli-Incalzi et al., they found that the turning point of HRQoL was connected to an FEV1 < 50% of what was predicted, which led to a marked decline [11], This result is consistent with our findings, which revealed a significantly lower utility scores in patients with an initial FEV1 of < 50% of prediction. However, a dynamic change in rapidly declining utility starting at 2 years of follow up, and the rate of decline was accelerated after 4 years of follow up had not been observed previously. Therefore, an FEV1 < 50% of prediction at baseline in older patients with COPD could be one of the predictors for the rapid and continuous decline of HRQoL. Both FEV1 < 50% of prediction and exacerbations requiring hospitalisation were contributing factors for the deterioration of HRQoL in this study. Jones et al. reported that HRQoL is associated with the severity of COPD and a history of disease exacerbation revealed a more impaired HRQoL [12]. An increased frequency of exacerbations was associated with a significantly worsened health status according to impaired SGRQ scores [7]. SAE history was associated with deterioration in HRQoL during 7 years of follow up in this study. Pulmonary rehabilitation improves dyspnea, exercise tolerance, and health status and reduces anxiety and depression for patients with COPD [9]. Moreover, early pulmonary rehabilitation (< 90 days after hospital discharge due to SAE) reduces the rate of readmission and mortality [13]. Therefore, early pulmonary rehabilitation for high-risk patients, such as FEV1 < 50% of prediction before 2 years after enrolment in this study, may prevent them from them entering the phase of a rapid decline of HRQoL. Increased comorbidities were also associated with poorer HRQoL. Specific concomitant diseases were related to a decreased HRQoL, such as cardiovascular disease, emotional problems, musculoskeletal disease, and diabetes [12, 14]. Comorbidity with CKD was one of the contributing factors for the deterioration of HRQoL in this study. The prevalence of CKD varied from 2 to 20% in patients with COPD according to different methodological approaches of analysis [15, 16]. The pathophysiological contribution of COPD in declined renal function might be due to hypoxemia, hypercapnia, and systemic inflammation. Additionally, cigarette smoking, and older age are also important risk factors for the development of CKD [17]. However, previous studies revealed that CKD had no significant effect on disease exacerbation or HRQoL for COPD [15, 18]. In this study, we found that CKD was an independent factor for declining HRQoL in older patients with COPD during long term follow up, which has not been reported.

The phase of rapid and continuous decline of HRQoL, initially in the usual activities and pain/discomfort domains, followed by the morbidity, self-care, and depression/anxiety domains approximately 2 and 4 years after enrolment, respectively, in this study.

### Usual activities

Free-living physical activities could reflect subjective lifestyle health. Compared to healthy control subjects, patients with COPD suffered from limited daily physical activities [19]. Patients with COPD had lower durations, intensities, and counts in daily physical activities than healthy subjects. However, the correlation between disease severity and daily physical activities was moderate, and not strong [19]. It could be possible because patients with COPD terminated physical activities due to skeletal muscle fatigue but not respiratory muscles [20]. A comprehensive pulmonary rehabilitation programme has a positive effect on greater participation in daily physical activity for patients with COPD by means of increased exercise capacity and adaptive behavioural change [21]. A continuous and accelerated decline in the usual activity domain of quality of life began early, before the second year of follow up in this study. Therefore, our findings support the importance of early pulmonary rehabilitation, especially for older patients with COPD with FEV1 < 50% of prediction.

### Pain/Discomfort

Chronic pain is common in patients with COPD, with prevalence ranging from 45 to 82% according to different studies [22–24]. The prevalence of pain was not correlated with the severity of airflow obstruction in COPD [23, 25]. Chronic pain has been linked to emotional problems with anxiety and depression in patients with COPD [26, 27]. Functional disabilities due to pain are also strongly associated with ageing [28]. Furthermore, impaired HRQoL and disease-specific health status were reported to be associated with chronic pain in patients with COPD [23, 29]. Patients with advanced COPD did not tend to suffer from pain more frequently than those with mild disease. However, pain intensity usually becomes worse with disease instability or deterioration of other clinical symptoms [30]. Therefore, patients in not only the severe stage but also the mild and moderate stages of COPD may suffer from chronic pain and discomfort early in the disease progression, as shown during the first 2 years of follow up in this study. This study also revealed that the intensity of pain or discomfort may continue to deteriorate after 2 years. However, this issue may be missed by clinicians because it does not always correlate with COPD severity and can be caused by other clinical symptoms [26, 29, 30]. Early detection and recognition of pain/discomfort in COPD may improve patients’ life quality.

### Mobility

Impaired pulmonary function was associated with mobility limitations in patients with COPD. For older patients with COPD, physical disabilities in carrying lighter objects, walking upstairs, and walking in the neighbourhood were related to reduced lung function (FEV1) rather than skeletal muscle strength [31]. Restricted Life-Space mobility was related to more severe dyspnea, elevated depressive symptoms, decreased quality of life, and disease exacerbation, and could also predict hospital readmission among patients with COPD [32, 33]. In the present study, the mobility domain of disease tracking revealed 3–4 years of a prolonged stable phase, which was followed by a phase of accelerated deterioration in the FEV1 < 50% group.

### Self-care

The capacity for self-care activities is impaired in patients with COPD [34]. Different self-care abilities were observed between healthy people and patients with COPD. This may be because more self-care techniques were needed for managing the clinical symptoms of COPD, including cough, phlegm, breathlessness, and fatigue [35]. Self-management interventions, including individualised education programmes and shared decision-making activities, could improve self-care behaviours [36]. Early self-management intervention before patients enter rapid deterioration of HRQoL (3–4 years after follow up in this study) may have better efficacy. Among these interventions for patients with COPD, energy conservation techniques are promoted to decrease clinical symptoms of dyspnea and fatigue, and this may also reduce energy expenditure while performing activities of daily living [37, 38]. 

### Anxiety/depression

The mechanism of emotional problems in COPD is as of yet undetermined [39]. However, clinical symptoms and impaired quality of life might be the determinants of depression in COPD [40]. For patients with stable COPD, the rate of comorbidity with depression ranged from 7–42% [41–43]. Patients with a higher COPD disease severity tend to suffer from depression more than those with mild disease [39]. Rapidly increasing anxiety and depression after 4 years of follow up was noted in this study. Moreover, the trend of increased anxiety and depression became increasingly severe during the long-term follow up period. Emotional problems with anxiety and depression in patients with COPD remain underdiagnosed and undertreated [44]. Adequate screening programmes for anxiety and depression could be the first step to obtaining an earlier and more accurate diagnosis [45]. Tailoring mental health treatments with the integration of pulmonary rehabilitation and pharmacological or non-pharmacological interventions, such as cognitive behavioural therapy and relaxation therapy, could reduce anxiety and depressive symptoms [46]. Personalised strategies could be implemented to address the mental health comorbidities through a multidisciplinary approach in patients with COPD.

### Limitations and strengths

This study has some limitations. First, the relatively small size of the patient cohort from one medical centre might have limited the generalisability. Second, few female participants were enrolled in this study, which was mainly because cigarette smoking is less prevalent among women (< 5%) in Taiwan [47]. 

The strength of this study is that it is the first research focusing on the dynamic changes in HRQoL in older patients with COPD over 7 years, offering long-term follow up on quality of life in multiple dimensions. Additionally, our results offer insight into the possible point of time at which the health status of different domains begins to rapidly decline. Such knowledge can help care providers implement suitable and timely interventions such as early pulmonary rehabilitation, energy conservation techniques, and medical therapy for anxiety and depression for older patients with COPD.

## Conclusions

An FEV1 of < 50% of prediction was a novel predictive factor for patients who progress to.

the phase of rapid and continuous decline of HRQoL, initially in the usual activities and pain/discomfort domains, followed by the morbidity, self-care, and depression/anxiety domains. SAE history and comorbidity with CKD were both contributing factors to the deterioration of HRQoL in long-term follow up. Future studies to evaluate the effect of early detection of high risks for rapid deterioration in older patients with COPD, along with appropriate interventions such as early pulmonary rehabilitation, were needed to improve their life trajectory.

## Electronic supplementary material

Below is the link to the electronic supplementary material.

Supplementary Material 1

## Data Availability

Full data sets are not currently available publicly to protect patient privacy. However, the data can be requested reasonably from the corresponding author.

## Abbreviations

COPD: Chronic Obstructive Pulmonary Disease
HRQoL: Health-related quality of life
EQ-5D: EuroQol Five-Dimension
FEV1: Forced expiratory volume in the first second
SAE: Severe acute exacerbation
BMI: Body Mass Index
mMRC: modified Medical Research Council
CAD: Coronary Artery Disease
CKD: Chronic Kidney Disease
LAMA: Long-Acting Muscarinic Antagonist
LABA: Long-Acting Beta-Agonist
ICS: Inhaled Corticosteroids
