# Supplementary material for: Dynamic changes in quality of life in older patients with chronic obstructive pulmonary disease: a 7-year follow up
Source: Health Qual Life Outcomes. 2024 Sep 11;22:76. doi: 10.1186/s12955-024-02296-1 (PMC11389236; doi:10.1186/s12955-024-02296-1)
Supplement: Supplementary file 1 — Supplementary Material 1 [file 12955_2024_2296_MOESM1_ESM.docx]

**Supplementary figure 1**

**Dynamic changes of utility values of quality of life in older COPD patients with different stages of pulmonary function between 1 March 2012 and 31 December 2019.**


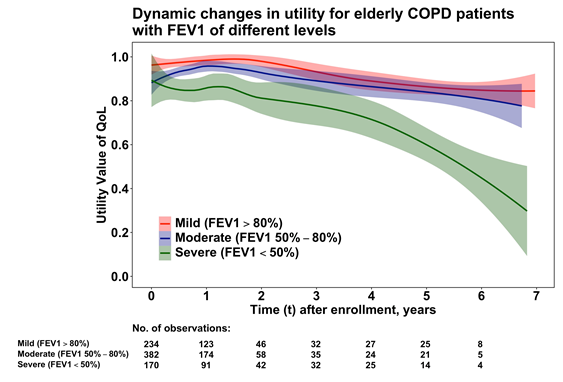


The dynamic change of utility values of EQ-5D-3L was analysed by a kernel smoothing method. Patients with FEV1 < 50% of prediction entered the phase of rapid declined of utility values approximately 2 years later and a more rapid decline was started approximately 4 years later from the beginning of follow-up.

**Supplementary figure 2**

**Dynamic changes of health domains in ‘usual activities’ (A) and ‘pain/discomfort’ (B) for older COPD patients with different stages of pulmonary function** **between 1 March 2012 and 31 December 2019.**

**(A)**


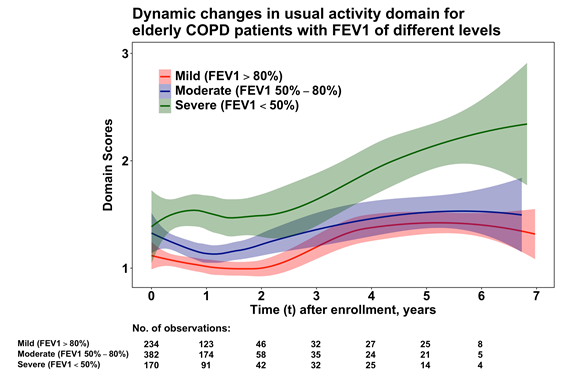


**(B)**


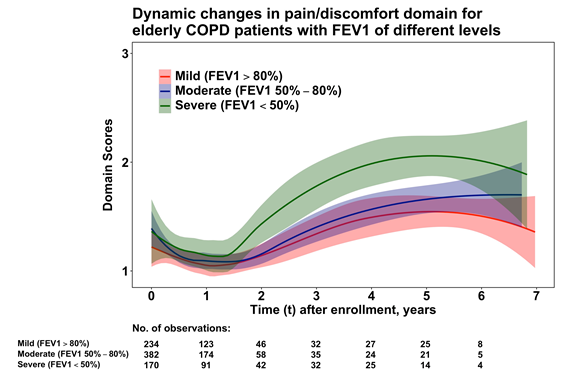


The dynamic change of domain scores of EQ-5D-3L was analysed by a kernel smoothing method. Distinctly worsened scores in patients with FEV1 < 50% of prediction were noticed at approximately 2 years later from the beginning of follow-up in the (A) ‘usual activity’ and (B) ‘pain/discomfort’ domains. The dynamic change of ‘pain/discomfort’ domain reached a plateau approximately 4 years later from the beginning of follow-up but continuous rapid progression of domain scores in the ‘usual activity’ domain.

**Supplementary figure 3**

**Dynamic changes of health domains in ‘mobility’ (A), ‘self-care’ (B), and ‘anxiety/depression’ (C) for older COPD patients with different stages of pulmonary function between 1 March 2012 and 31 December 2019.**

**(A)**

**
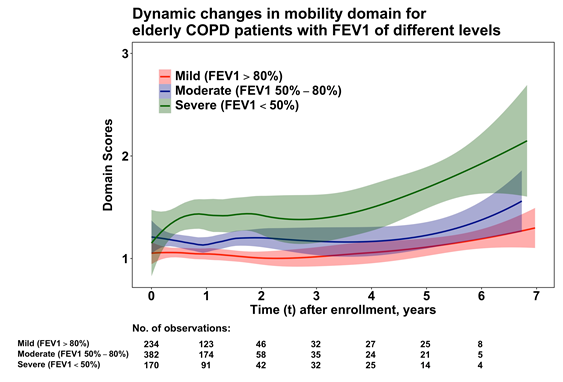
**

**(B)**


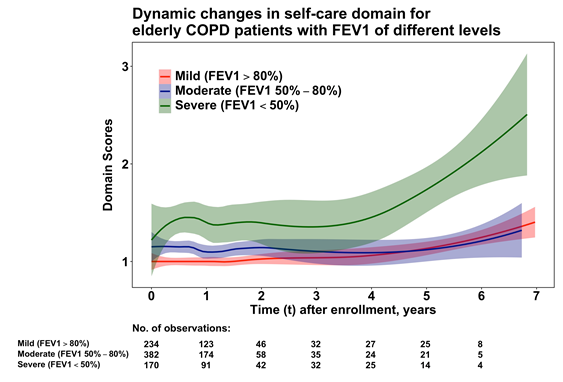


**(C)**

**
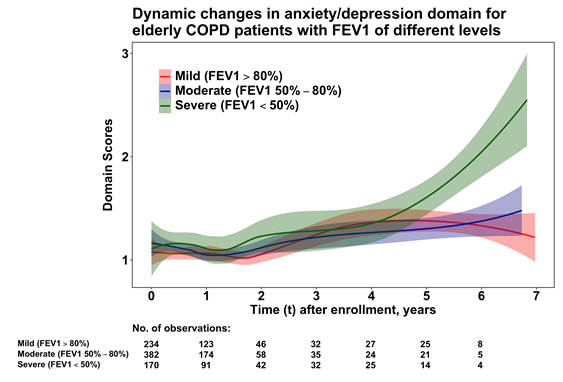
**

The dynamic change of domain scores of EQ-5D-3L was analysed by a kernel smoothing method. Distinctly worsened scores in patients with FEV1 <50% of prediction were noticed at approximately 4 years later from the beginning of follow-up in the (A) ‘mobility’, (B) ‘self-care’ and (C) ‘anxiety/depression’ domains.
